# Effect of ferritin, INR, and D-dimer immunological parameters levels as predictors of COVID-19 mortality: A strong prediction with the decision trees

**DOI:** 10.1016/j.heliyon.2023.e14015

**Published:** 2023-03-05

**Authors:** Mehmet Tahir Huyut, Zübeyir Huyut

**Affiliations:** a Erzincan Binali Yıldırım University, Faculty of Medicine, Department of Biostatistics and Medical Informatics, Erzincan, Turkey; b Van Yuzuncu Yıl University, Faculty of Medicine, Department of Biochemistry, Van, Turkey

**Keywords:** COVID-19, Mortality risk biomarkers, Coagulation tests, Immunological tests, Ferritin, CHAID decision Trees, Machine learning, Artificial intelligence

## Abstract

**Background and objective:**

A hyperinflammatory environment is thought to be the distinctive characteristic of COVID-19 infection and an important mediator of morbidity. This study aimed to determine the effect of other immunological parameter levels, especially ferritin, as a predictor of COVID-19 mortality via decision-trees analysis.

**Material and method:**

This is a retrospective study evaluating a total of 2568 patients who died (n = 232) and recovered (n = 2336) from COVID-19 in August and December 2021. Immunological laboratory data were compared between two groups that died and recovered from patients with COVID-19. In addition, decision trees from machine learning models were used to evaluate the performance of immunological parameters in the mortality of the COVID-19 disease.

**Results:**

Non-surviving from COVID-19 had 1.75 times higher ferritin, 10.7 times higher CRP, 2.4 times higher D-dimer, 1.14 times higher international-normalized-ratio (INR), 1.1 times higher Fibrinogen, 22.9 times higher procalcitonin, 3.35 times higher troponin, 2.77 mm/h times higher erythrocyte-sedimentation-rate (ESR), 1.13sec times longer prothrombin time (PT) when compared surviving patients. In addition, our interpretable decision tree, which was constructed with only the cut-off values of ferritin, INR, and D-dimer, correctly predicted 99.7% of surviving patients and 92.7% of non-surviving patients.

**Conclusions:**

This study perfectly predicted the mortality of COVID-19 with our interpretable decision tree constructed with INR and D-dimer, especially ferritin. For this reason, we think that it may be important to include ferritin, INR, and D-dimer parameters and their cut-off values in the scoring systems to be planned for COVID-19 mortality.

## Highlights

• All immunological parameters were significantly higher in the non-surviving COVID-19 patients.
• Our interpretable decision tree was built with only ferritin, INR and D-dimer.
• Our decision tree predicted survivors (99.7%) and deaths (92.7%) highly accurately.
• The range of ferritin (389.0 μg/L -395.0 μg/L) was important in COVID-19 mortality.
• The value of D-dimer (<1239.140 μg/L) was remarkable in COVID-19 mortality.

## 1 Introduction

COVID-19 is an infectious disease caused by SARS-CoV-2 infection. Since the World Health Organization (WHO) declared the SARS CoV2 infection as a pandemic, the epidemic still maintains its severity [1,2]. Previous studies showed that this disease can accompany multi-organ dysfunction and cause a range of symptoms [1,3,4].

While many studies indicate that COVID-19 disease may be asymptomatic, it has also been reported that the disease may be associated with severe ARDS, which is thought to be due to inflammation [[4], [5], [6]]. In addition, Chalmers et al. [7] pointed out that the most important cause of coronavirus deaths is the intense and unbridled release of proinflammatory cytokines. However, efforts to define and treat the relationship structure between COVID-19 disease and hyperinflammation continue [8]. Additionally, some studies have highlighted that COVID-19 may be a manifestation of broader hyperinflammatory conditions such as secondary hemophagocytic lymphohistiocytosis (sHLH) that can be characterized by cytokine release syndrome (CRS) [[8], [9], [10], [11]]. In particular, one of the most important features of these syndromes is hyperferritinemia [8]. Ferritin, known as the essential intracellular iron storage protein, is a positive acute phase reactant that rises in many inflammatory conditions [12,13]. In addition, it was stated that very high ferritin levels may cause diseases such as macrophage activation syndrome and septic shock [8,13]. Similarly, many studies have shown the importance of the immunomodulatory effect of ferritin in mortality and inflammatory processes [8,[12], [13], [14]]. In addition, it has been reported in many studies that ferritin may be a direct mediator of the immune system as a signal molecule and its distinctive feature in hyperferritinemia syndromes has been emphasized [7,12,[15], [16], [17]].

High levels of ferritin, D-dimer, lactate dehydrogenase, and IL-6 were reported to be an indicator of poor prognosis and risk of COVID-19 mortality [11,12,18]. Efforts to treat COVID-19 have included testing various anti-inflammatory biological agents to inhibit this potent immune response [12,19,20]. Kurniawan et al. [21] reported that hyper inflammation, coagulation cascade, multi-organ failure, which play a role in the etiopathogenesis of COVID-19, and biomarkers such as CRP, D-Dimer, LDH, and albumin associated with these conditions may be useful in predicting the outcome of COVID-19.

In a meta-analysis, Hariyanto and Kurniawan [22] found a high correlation between obstructive sleep apnea (OSA) and poor outcomes of COVID-19 patients and stated that OSA increases the mortality of the disease. Hariyanto et al. [23] found a high level of association between Epilepsy and increased severity of COVID-19 and death from COVID-19 in a meta-analysis. Hariyanto et al. [24] found in a meta-analysis that Dementia is associated with advanced and fatal COVID-19 infection. Severe COVID-19 causes hypoxia, which increases the risk of thrombosis due to increased blood viscosity and hypoxia-inducible transcription factor-dependent signaling pathways [21].

In recent years, we see that artificial intelligence technologies, which have been used with great success in many fields, are increasingly used in the diagnosis and prognosis of diseases and the improvement of treatment processes, especially in the field of medicine [25]. The most important reason for this is the power of machine learning (ML) algorithms, which is considered an important part of artificial intelligence (AI) technologies, to reveal the hidden relationships (machine learning - feasibility) between patterns [2,26,27]. In this context, ML approaches, also applied to medical science issues, are rapidly being developed due to their high performance in predicting outcomes, reducing drug costs, improving patient health, and making real-time decisions to improve healthcare value and quality [[28], [29], [30]].

The artificial intelligence-based on chi-squared automatic interaction detection (CHAID) method is frequently used in various disciplines to predict how some variables affect other variables, as well as other early applications in medical and psychiatric research [34]. Decision tree methods use multipath splits as an assumption. In addition, in studies with small sample sizes, the level of reliability of the answered groups decreases rapidly [35]. Therefore, a large sample is needed for decision trees to work effectively. An important advantage of CHAID analysis according to the alternative methods such as multiple regression is that it is a non-parametric method [36]. Therefore, it is resistant to problems with multicollinearity, outliers, distribution, structure, and missing data [37]. CHAID analysis is a method that can model categorical and continuous variables together and provides highly accurate classification or estimation performance in large samples [28,38]. With this method, the relationships between the independent variables are detailed, and easy, understandable, and interpretable outputs in the form of trees can be obtained even in the most complex models [28]. In addition, since variable combinations are also examined in this method, interactions can be also evaluated. Decision tree inductors are algorithms that automatically generate a decision tree from a dataset. This algorithm aims to obtain the optimal decision tree by minimizing the generalization error. However, it is important to define other target functions that affect the performance of the decision tree and to determine the number of nodes and the average depth. The induction of an optimal decision tree from data is considered a difficult task [39].

Previous AI studies did not use many of the immunological parameters to predict mortality from COVID-19 and reported relatively lower classifier performance than this study [2,3,5,6]. Moreover, previous studies [9,[18], [19], [20],^25^,[31], [32], [33]] have generally focused on the early detection of COVID-19 disease and have addressed relatively small samples. In addition, artificial intelligence studies that predict the mortality of the disease based only on immunological parameters and detect patients with a high probability of dying in the early phase are insufficient.

In particular, the usefulness and effective breakpoints of ferritin and D-dimer in predicting COVID-19 mortality have not yet been fully established [7,12,32,33]. Therefore, the predictive role of ferritin level as a pro-inflammatory factor in the uncontrolled cytokine storm at risk of poor outcome in COVID-19 patients needs further confirmation [7,11]. In addition, we found that a limited number of studies have been conducted on the effect of ferritin in the prediction of COVID-19 mortality and that these studies cover the early stages of the epidemic. In addition, the statistical power of the predictive indicators for the cut-off values of ferritin, which was used in the detection of severe patients in previous studies, was relatively lower than in this study.

In this study, it was determined which immunological parameters are the most important source of variation affecting COVID-19 mortality with the CHAID decision tree from ML models. In this context, important critical levels of other immunological parameters especially serum ferritin, which affects COVID-19 mortality, were calculated and their importance was interpreted to detect surviving and non-surviving patients and to monitor the poor prognosis of the disease. Accordingly, the success of our interpretable decision tree, which was created only with the immunological values in this study, in the detection of surviving and non-surviving patients was calculated. We think that this study will help in the identification of severely infected COVID-19 patients with a high probability of death and will make important contributions to the determination of the poor prognosis of the disease.

## 2 Material and Method

### 2.1 1. Participant's criteria, study design

The data used in this retrospective cohort study were collected digitally from the Erzincan Binali Yıldırım University Mengücek Gazi Training and Research Hospital information system between August and December 2021, and the data conforming to our criteria were included in the study. The data used in this study includes the information of 2568 patients who were hospitalized and treated only with the diagnosis of COVID-19 between the specified dates. Patients who were not diagnosed with COVID-19 on the specified dates and were less than 18 years old were excluded from the study. In our hospital, COVID-19 was diagnosed in cases with SARS-CoV-2 detected only in nasopharyngeal or oropharyngeal swabs by RT-PCR. Age and gender characteristics of detected COVID-19 patients were recorded. Immunological test records of these patients from admission to discharge, as well as mortality and survival, were examined. The data of the patients were divided into two groups according to their exit information: those who died from COVID-19 (n = 232), and those who recovered from COVID-19 and survived (n = 2336). Patients who died from COVID-19 were defined as severely ill, of whom 90% were intensive care patients. Since our study was a retrospective scan, the comorbidity data of most of the patients could not be accessed.

In this study, demographic data and immunological blood values of patients who died and survived COVID-19 were compared first. Then, CHAID analysis, which was one of the ML decision trees methods, was used to determine the effectiveness of the immunological blood values in estimating the mortality of COVID-19. Our interpretable decision tree obtained via CHAID analysis identified the most important immunological parameters influencing COVID-19 mortality. In addition, the cut-off values of the most effective parameters in the estimation of the mortality of the disease were calculated and the relationship structure between the parameters was examined. The complexity matrix was used to evaluate the performance of the decision tree. According to this matrix, the success of the obtained decision tree in predicting patient groups was calculated. In addition, the changes in the ferritin, INR, and D-dimer parameters used in the construction of our decision tree in the following days during the treatment process were recorded. These values of the patient groups were calculated as average and were examined until the exit of the patients.

### 2.2 Immunological routine laboratory parameters used in the study

C-reactive protein (CRP), D-dimer, ferritin, fibrinogen, international normalized ratio (INR), Prothrombin time (PT), Procalcitonin (PCT), erythrocyte sedimentation rate (ESR), Troponin, and activated partial prothrombin time (aPTT) values measured at admission to the hospital were included in the study. Ferritin was assessed by an immunoassay of chemiluminescence (Centaur XP, Siemens Healthcare, Germany). Prothrombin time (PT), activated partial prothrombin time (aPTT) and fibrinogen were determined with a completely digital coagulation instrument of Ceveron- Alpha (Diapharma Group Inc., West Chester, Canada). C-reactive protein (CRP) was measured with the nephelometric method on BNTM II System (Siemens, Munich, Almanya). Procalcitonin (PCT), D-dimer, and Troponin I were analyzed from the whole blood on the AQT90 flex RadiometerVR (Bronshoj, Denmark). The erythrocyte sedimentation rate (ESR) was measured using a TEST 1 BCL device (Alifax, Padova, Italy) based on the principle of photometric capillary flow kinetic analysis.

### 2.3 Data extraction and workflow

**Fig. 1:**
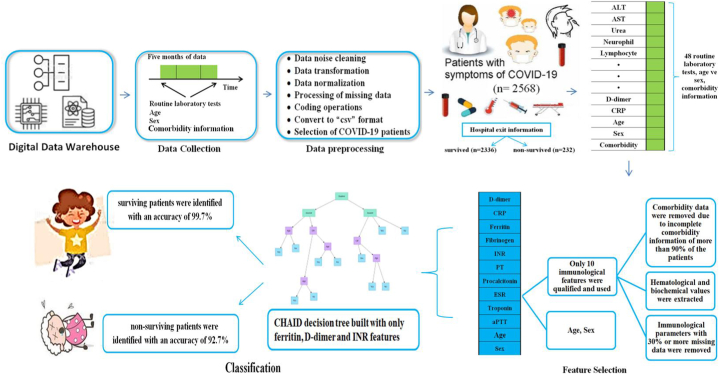
Work flow chart of this study.

Between the specified dates, the diagnostic criteria of 80 thousand patients were examined in our hospital registry system and the patients who were diagnosed with COVID-19 as a result of the RT-PRCR test were filtered. In the registry, there were about 50 routine blood values (RBV), exit information, gender, age, and various underlying suspected disease diagnoses of COVID-19 patients. The comorbidity data of patients diagnosed with COVID-19 were analyzed. However, these data were not used in this study, as comorbidity data for these patients was largely missing (more than 90%). Hematological and biochemical routine blood values were not included in the study because they did not comply with the concept of the study. Outliers in the data set were normalized by filling the missing data in the recorded immunological parameters with the average of the relevant parameter distribution. We abandoned features with 30% or more missing data from the recorded immunological parameters, and only 10 features were qualified and used in the study. Ultimately, in our dataset, patients who lived and died from COVID-19 had 10 immunological test results, age, and gender. The workflow of this study is summarized in Fig. 1.

### 2.4 Statistical analysis

Categorical variables were expressed as frequency and percentage, while median and quartile values of continuous variables were presented. The Shapiro-Wilk test was used to confirm the normality of the distributions of quantitative variables. In the comparison of continuous variables, normally distributed variables were analyzed with the independent sample *t*-test, and those that were not normally distributed were analyzed with the Mann-Whitney *U* test. Categorical variables were analyzed with the χ2 test. CHAID analysis, one of the ML decision tree methods, was used to evaluate the predictive immunological parameters in the estimation of the mortality of COVID-19 disease. SPSS (version 20.0, SPSS Inc, Chicago) package programs were used for statistical analysis of the data. p < 0.05 was considered statistically significant. CHAID analysis, one of the ML decision tree methods, was used to collectively evaluate the predictive immunological parameters in the estimation of the mortality of COVID-19 disease.

The following statistical model specifications and stopping criteria were applied in the CHAID decision tree analysis:

(1) Significant level for separation nodes was determined as p < 0.05.
(2) The Bonferroni method was used to obtain significant correction values.
(3) The minimum change in expected cell frequencies was 0.001.
(4) Pearson χ2 independence test was used to determine the relationships between independent (explanatory) variables.
(5) Model depth is set to maximum.
(6) A 10-fold cross-validation was used to evaluate the tree structure and
(7) Misclassification risk was calculated as a measure of model reliability.

Decision trees are considered one of the most popular approaches to representing classifiers. Comprehensive CHAID model estimation starts with the entire sample (called “parent node/root”) and then divides the master nodes into meaningful homogeneous subgroups (“child nodes/leaf”) according to a certain discrete function. Splitting continues until predetermined stopping criteria are met. Each leaf is assigned a class that represents the most appropriate target value. Specimens are classified according to the results of the tests by going through all the leaves from the root of the tree.

This algorithm uses the χ2 independence test to determine the relationships between the independent (explanatory) variables and then selects the explanatory variables that best explain the dependent (response) variable based on the “IF–THEN” logic. To obtain significant correction values, CHAID analysis using the Bonferroni test can be used for prediction (similar to regression analysis), classification, as well as detection of interactions between variables [35,40].

## 3 Results

### 3.1 Demographic characteristics and immunological tests

The demographic characteristics of patients who died from COVID-19 or survived after treatment and the immunological blood values of all patients at admission to the hospital are shown in Table 1. While 2336 (90.9%) of 2568 patients in this study survived, 232 (9.1%) died. While 142 (61.2%) of the patients who lost their lives were male, 90 (38.8) were female. While the mean age of the surviving patients was 55.00, it was 76.00 for the patients who died.

**Table 1:** Comparison of inflammatory/immunological, cardiac and coagulation variables between survivor and non-survivor groups.

		**Survivor Covid-19**	**non-Survivor Covid-19**	
**Sex**				*p-value
Male n (%)		1148 (49.1)	142 (61.2)	<0.001
Female n (%)		1188 (50.9)	90 (38.8)	
		**Median (IQR)**	**Median (IQR)**	**p-value
Age		55.00 (30.00)	76.00 (14.00)	<0.001
**Parameters**	**Reference range**			
CRP (mg/L)	0–5	6.76 (22.18)	72.00 (55.80)	<0.001
D-dimer (μg/L)	80–583	541.00 (591.95)	1277.00 (177.00)	<0.001
Ferritin (μg/L)	22–322	225.95 (170.40)	395.00 (29.5)	<0.001
Fibrinogen (g/L)	1.8–4.5	3.211 (0.77)	3.50 (0.05)	<0.001
INR	0.6–1.2	1.10 (0.13)	1.25 (0.24)	<0.001
PT (Sec)	10.1–15.9	13.10 (1.50)	14.83 (14.20)	<0.001
Procalcitonin (μg/L)	<0.15	0.12 (0.04)	2.75 (0.47)	<0.001
ESR (mm/hr)	0–20	17.00 (31.00)	47.05 (29.00)	<0.001
Troponin (ng/L)	10–23	17.00 (12.51)	57.00 (102.75)	<0.001
aPTT (Sec)	22–45	35.34 (7.85)	31.00 (2.00)	<0.001

CRP: C-reactive protein; INR: international normalized ratio; PT: prothrombin time; ESR: erythrocyte sedimentation rate; aPTT: activated partial prothrombin time; The **p values showing the differences of the parameters show the Mann-Whitney *U* test result. The *p value showing the difference of gender shows the result of the Chi-square test. p < 0.05 was considered significant.

Chi-square test results and gender were determined to be associated with disease mortality (p < 0.001). According to the results obtained, the male gender was seen as a factor in increasing mortality. In addition, the age of the patients who died was significantly higher than the patients who survived (p < 0.001). Accordingly, advanced age was found to be associated with the mortality of the disease (Table 1).

When Table 1 is examined, patients who died from COVID-19 had C-reactive protein (CRP, 72.00 against 6.76 x mg/L), D-dimer (541.0 against 1277.00 x μg/L), Ferritin (225.95 against 395.0 x μg/L), Fibrinogen (3.21 against 3.50×*g*/L), international normalized ratio (INR, 1.10 against 1.25), PT (13.10 against 14.83 s), procalcitonin (0.12 against 2.75 x μg/L), erythrocyte sedimentation rate (ESR, 17.00 against 47.05 x mm/hr) and troponin (17.00 against 57.00 x ng/L) values were higher than in the living patients. was high. However, activated partial prothrombin time (aPPT, 35.34 against 31.00 x sec) in deceased patients was lower than in surviving patients.

### 3.2 CHAID decision tree for detecting surviving and non-surviving from COVID-19

**Fig. 2:**
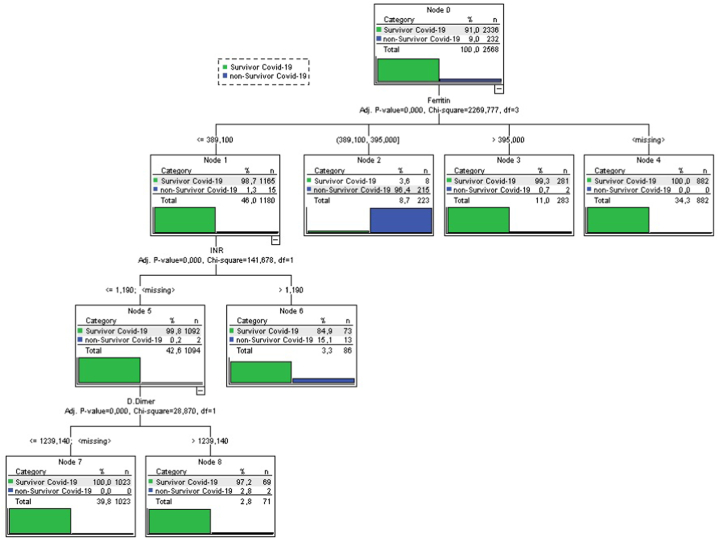
Decision tree structure and cut-off values of predictive immunological variables affecting COVID-19 mortality.

The CHAID decision tree model created to determine the predictive immunological variables affecting the mortality of COVID-19 patients was presented in Fig. 2. These values were the values measured at admission to the hospital. When the decision tree diagram in Fig. 2 is examined, 232 (9.0%) of 2568 patients included in the study died, while 2336 (91.0%) of them recovered. As seen in Fig. 2, the decision tree diagram estimating the mortality of COVID-19 patients consisted of only the immunological parameters ferritin, INR, and D-dimer. The presence of ferritin and subsequently INR and D-dimer at the root of the decision tree can be interpreted as the robustness of the approach to estimating disease mortality and indicates the clinical accuracy of our decision tree.

When the decision tree was examined, it was seen that the variable with the most important effect on the mortality of the disease was “ferritin” (χ2 = 2269.777 and p < 0.001). The patients were divided into three different groups according to the ferritin parameter, whose cut-off values were determined for the estimation of the mortality of COVID-19. Also, the ferritin value could not be reached in 34.3% (n = 882) of the patients included in the study. However, those were living patients. Accordingly, the first group had ferritin values < 389,100 μg/L and the mortality of this group was 1.3%. The second group had ferritin values in the range of 389,100 μg/L to 395,000 μg/L and the mortality of this group was 96.4%. The third group had ferritin values > 395.00 μg/L and the mortality of this group was 0.7%. According to these results, when the ferritin value of the third group is not taken into account, it can be said that there is a great increase in the mortality rate with the increase in the ferritin value compared to the first two groups (1.3% vs. 96.4%). Since the first two groups constitute the majority of the study, according to these data, we can say that the increase in ferritin values, in general, induces COVID-19 mortality. Although the third group's values seem to contradict the values of these two groups, the findings do not contradict each other. Because these data are the values measured at admission to the hospital, there was probably a decrease in the ferritin values of most of the patients living in the third group in the following days.

In patients with ferritin values less than 389.100 μg/L according to the decision tree, the most important immunological parameter affecting mortality was INR (χ2 = 141.668 and p < 0.001). Patients were divided into two groups according to the cut-off values of INR. Mortality of patients with ferritin less than 389,100 μg/L and INR= ≤ 1190 were 0.2%, while patients with an INR = > 1.190 had a mortality at 15.1%. According to these results, it can be said that increasing the INR value induces mortality (15.1% against 0.2%).

When the decision tree was analyzed, D-dimer was the most effective immunological parameter in predicting mortality in patients with ferritin less than 389,100 μg/L and INR= ≤ 1190 (χ2 = 28,870; p < 0.001). Patients were divided into two groups according to the cut-off values of D-dimer (≤1239.140 μg/L, >1239.140 μg/L). In addition, patients with ferritin= <389,100 μg/L, INR= ≤ 1190 and D-dimer= ≤ 1239,140 μg/L values were all alive, whereas a mortality of patients with high D-dimer (>1239.140 μg/L) was 2.8%. According to this result, it can be said that increasing the D-dimer level induces mortality (0.8% vs. 2.8%).

Within the scope of the study, in detecting patient groups, the overall accuracy (success rate) of our decision tree model, which was created with Ferritin, INR, and D-dimer immunological variables to predict the mortality of COVID-19 was found at 99.0% and this data was statistically significant. (Fig. 2, Table 2). Accordingly, our decision tree model correctly classified 99.7% of the patients who survived and 92.7% of those who died, taking into account the cut-off values of INR and D-dimer parameters, especially ferritin. In addition, detecting surviving and non-surviving patients with a sensitivity and specificity value of over 90% showed that our decision tree is extremely robust and works with high performance even in unbalanced datasets (Table 3).

**Table 2:** The success of the CHAID decision tree created with the immunological parameters of ferritin, INR and D-dimer in the detection of patients who died and survived from COVID-19 disease.

Observed	Predicted		
	Survivor COVID-19	non-Survivor COVID-19	Percent Correct
Survivor COVID-19	2328	8	99.7%
non-Survivor COVID-19	17	215	92.7%
Overall Percentage	91.3%	8.7%	99.0%

**Table 3:** Classification performance criteria of the CHAID decision tree.

Statistic	Value	95% CI
Sensitivity/Recall	92.67%	88.53%–95.67%
Specificity	99.66%	99.33%–99.85%
Positive Predictive Value/Precision	96.41%	93.08%–98.17%
Negative Predictive Value	99.28%	98.86%–99.54%
Total Accuracy	99.03%	98.57%–99.37%

**Fig. 3:**
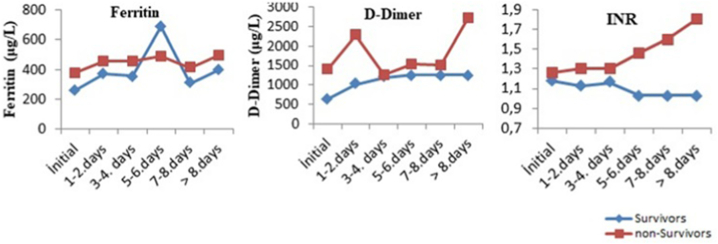
Dynamic changes of three immunological parameters that were effective in the prediction of COVID-19 mortality in the thirty days after hospitalization.

In addition, the changes in the ferritin, INR, and D-dimer parameters used in the construction of our decision tree on the days after hospital admission were presented in Fig. 3 according to the patient groups. Accordingly, Ferritin (except 5 and 6 days), D-dimer, and INR levels were higher in the group that did not survive admission to the hospital until discharge in the following days compared to the living group. However, it was remarkable that ferritin values were higher in patients living on the 5th and 6th days (Fig. 3).

## 4 Discussion

COVID-19 has attracted attention for its higher infectivity and mortality than influenza [1,3,41]. The majority of infected patients had mild symptoms. However, the prevalence of symptoms such as severe pneumonia, acute respiratory distress, and multi-organ dysfunction among infected patients was much lower [17,38,42]. In addition, the need to determine the immune status of especially risky patients and to determine biomarkers that affect the mortality of the disease continues [1,43]. Therefore, early detection of patients who require special care and with high death expectancy and effective identification of relevant biomarkers are important in terms of reducing the mortality of the disease [11,28,36,38].

In this study, the importance of monitoring immunological parameters by using decision trees in predicting the mortality of COVID-19 at acceptance and during hospitalization was evaluated. Our decision tree which was constructed with INR, D-dimer, and especially ferritin, perfectly predicted the mortality of COVID-19 (Fig. 2). In addition, the presence of ferritin and subsequently INR and D-dimer at the root of our decision tree revealed to support the clinical accuracy of our interpretable decision tree for estimating COVID-19 mortality.

In this study, 91% (n = 2336) of COVID-19 patients survived, while 9% (n = 232) of patients died. While 61.2% (n = 142) of the patients who died were male, 38.8% (n = 90) of them were female. While the mean age of the patients who survived was 55.00, it was 76.00 years for the patients who died (Table 1). According to the results, male gender and advanced age were found to be demographic characteristics that increase mortality (Table 1). Zhou et al. [18] found the COVID-19 mortality to be 28.3%, while Guan et al. [44] found it to be 1.4%. In other studies, mortality from COVID-19 was found to be 45%, 4.3%, and 28.4% [36,45,46]. The different mortality outcomes of COVID-19 may have resulted from the different sample sizes and case inclusion criteria used in the studies. Also, in this study, the increase in the severity and mortality of the disease by high age and male gender were found to be compatible with the literature [18,19,36,45].

Banerjee et al. [47] found the most successful glmnet model (92% sensitivity, 91% accuracy) in detecting patients among covid positive (n = 81) and healthy individuals (n = 517) using 14 RBV values. Huyut [48] ran 11 supervised machine learning models and used 28 RBV values to detect ICU patients and non-ICU patients in his study to determine the prognosis of COVID-19. In that study, Huyut [48] found the most successful models in identifying mildly infected patients, the local weighted learning model with 0.95%-AUC and the Kstar model with 0.91%-AUC. Brinati et al. [49] used 13 RBV features for the detection of COVID-19 with various classification models, and the random forest and logistic regression model were the models with the highest accuracy (82% and 78%, respectively). Huyut and Velichko [50] operated 51 RBV values with the LogNNet deep neural network model to quickly, economically and safely detect the diagnosis and prognosis of COVID-19 disease. They achieved 99% accuracy in the diagnosis of the disease and 83% accuracy in the determination of the prognosis of the disease. Zhang et al. [51] used various demographic and RBV with LASSO-based artificial neural network (ANN) models with the least absolute shrinkage and operator of choice (LASSO) regression to identify predictors of COVID-19 mortality. The success of the LASSO regression model and the LASSO-based ANN model that was run with the 9 prognostic factors they identified for mortality, in determining the clinical status of patients was 98%-AUC [51].

Zhu et al. [52] applied a deep learning algorithm to predict the mortality of COVID-19 by using 78 features from demographic, clinical, and laboratory tests. They found the method's performance in estimating COVID-19 mortality as 95.4%-AUC [52]. Alle et al. [53] applied 70 features from clinical and laboratory tests to various machine learning models to predict COVID-19 mortality, and XGboost and logistic regression models performed best in mortality prediction, respectively (83%-AUC vs 92%-AUC). In addition, Alle et al. [53] found that serum ferritin was the most important predictor of COVID-19 mortality, via the community-averaged Bayesian network. For the prediction of critical COVID-19 patients, Gao et al. [54] developed an ensemble model derived from a Support Vector Machine (SVM), Gradient Augmented Decision Tree (GBDT), and Neural Network (NN) algorithms by using immune-inflammatory properties [54]. They found AUC-0.99 the success of the developed model in detecting severely infected patients. Vaishnav et al. [55] used various machine learning models to predict COVID-19 deaths in the country of India, and the decision tree regression model produced an accuracy of 70% and the random forest regression model produced an accuracy of 76%. Booth et al. [56] developed a machine learning model using five routine laboratory parameters to identify prognostic serum biomarkers in COVID-19 patients at risk of death, and the model predicted mortality with 91% sensitivity and 91 specificity. Kukar et al. [57] created a machine-learning model to diagnose 5333 negative and 160 COVID-19-positive patients with various bacterial and viral infections based on routine blood tests. They found the classification success of the model as 81.9% sensitivity and 97.9% specificity [57]. In a study using tomography data and some RBV features, Mei et al. [58] proposed a model combining CNN and multilayer sensors and found the success of the model in diagnosing COVID-19 with 84% sensitivity and 83% specificity. In a study was conducted for the diagnosis of COVID-19, Soares [59] proposed a model combining SVM, ensembling and SMOTE Boost models which was using 15 RBV parameters, and he found the success of the model in diagnosis with 86% specificity, 70% sensitivity. In another study conducted for the diagnosis of COVID-19, using various RBV parameters, Soltan et al. [60] found the XGBoost method to be the most successful model with 85% sensitivity and 90% accuracy. Velichko et al. [61] studied a large patient population with 13 different ML classifier models to detect COVID-19, and the histogram based gradient boosting (HGB) model was the most successful, detecting all patients with 100% accuracy. Also, in that study, they implemented the internet of things (IoT) in online and offline mode for the diagnosis of COVID-19. Huyut et al. [62] used 16 different classifier models and RBV values on patients who died (n = 233) and recovered (n = 2364) from COVID-19 to detect important biomarkers that affect the mortality of COVID-19 disease and to determine the risk levels of these features. The most successful model in predicting the mortality of the disease with only three features (D-dimer, ESR, Direct Bilirubin) was the histogram based gradient enhancement (HGB) model with precision >0.98 and recall >0.98 [62].

Huyut and İlkbahar [38] used the CHAID decision tree algorithm to detect COVID-19 positive and negative patients and severely and mildly infected COVID-19 patients by using various biomarkers. They found that the performance of the decision tree had an 81.6% in determining the diagnosis of the disease and 93.5% in determining the prognosis of the disease as accuracy rate [38]. Huyut and Üstündağ [28] used the CHAID decision tree algorithm to detect COVID-19 positive and negative patients and severely and mildly infected COVID-19 patients using various blood gas parameters. They found the performance of the decision tree to be 68.2% in determining the diagnosis of the disease and 65.0% in determining the prognosis of the disease and an accuracy rate [28]. Using various demographic and clinical features, Doğanay et al. [36] determined the mortality of COVID-19 by using the CHAID decision algorithm with an accuracy rate of 81.0%. Al-Najjar et al. [63] used various machine-learning algorithms to detect patients who recovered and died from COVID-19. They found the accuracy of the CHAID decision tree algorithm to be 82.0% for the training set and 78.0% for the test set [63]. Guerrero et al. [64] examined the compliance of Canadian children and youth with 24-h movement rules during the COVID-19 outbreak using the CHAID algorithm in four different categories. They found accuracy rates ranging from 70% to 97.4% with this algorithm [64]. Feigin et al. [65] identified age-related biomarkers by using the CHAID algorithm to predict in-hospital mortality in COVID-19 patients. Kazawa et al. [66] identified characteristics of early-career nurse researchers who were adversely affected during the COVID-19 epidemic using the CHAID algorithm. Sacco et al. [67] identified the symptoms and chronological aspects of the spread of the SARS-CoV-2 virus among both residents and caregivers in a nursing home by using the CHAID algorithm. Seddawy and Mohamed [68] analyzed the impact of COVID-19 on e-commerce and online shopping by using the CHAID decision tree. They suggested that the CHAID algorithm can be used to determine the customer's purchasing preference, with a sensitivity of 89.09% and a classification error of 10.9% [68].

Ferritin has been suggested to be a useful marker for predicting patient outcomes in patients with COVID-19 [12]. Numerous studies are showing that higher ferritin levels, along with other proinflammatory markers, including CRP and IL-6, are associated with worse outcomes [12,18,69]. In another study, high levels of inflammatory markers such as CRP, erythrocyte sedimentation rate, IL-6, and procalcitonin in COVID-19 patients were reported to indicate hyperinflammatory reactions in COVID-19 patients [11]. Gandini et al. [70] noted an extremely elevated ferritin level (mean serum ferritin >500 μg/L) in all patients at the time of admission of severe COVID-19 patients, and reported that milder cases had a mean ferritin level of 303 ± 224 μg/mL. In addition, Gandini et al. [70] and Gao et al. [71] noted that the ferritin level of severe and intensive care COVID-19 patients was 2.6-fold and 5.8-fold higher than in mild patients, respectively. Dahan et al. [72] observed higher ferritin levels in middle to heavly COVID-19 patients than in slightly patients (mean ferritin level in mild patients 327.27 ng/mL, in middle patients 1555 ng/mL, in heavly patients 2817.6 ng/mL). A meta-analysis of data from 57563 COVID-19 patients and 189 observational studies reported a significant difference in mean ferritin levels between survivors and died [73]. Another meta-analysis involving 5350 patients showed that high ferritin led to a poor outcome in COVID-19 and was associated with the development of ARDS [74]. Taneri et al. [73] stated that plasma exchange, high-volume hemofiltration, and desferrioxamine could be used to reduce ferritin levels in COVID-19 patients. In addition, some studies reported that these therapies are currently used in the treatment of sepsis and macrophage activation syndrome [[74], [75], [76]]. In this study, patients who died from COVID-19 had 1.75 times higher ferritin values than patients who survived. In addition, the overall accuracy of the CHAID decision tree created to identify the predictive immunological variables affecting the mortality of COVID-19 patients in this study was 99% in classifying all patients (Table 2). Moreover, our decision tree correctly predicted 99.7% of living patients and 92.7% of deceased patients. Furthermore, detecting surviving and non-surviving patients with a sensitivity and specificity value of over 90% showed that our decision tree is extremely robust and works with high performance even in unbalanced datasets (Table 3).

In our interpretable decision tree, ferritin was found to be the most important immunological parameter for the mortality of COVID-19 (Fig. 2). In addition, patients with ferritin values of 389,10 μg/L to 395,00 μg/L had a significantly higher mortality rate than patients with ferritin values less than 389,10 μg/L (96.4% vs. 1.3%). These results showed that elevated ferritin values significantly increased mortality. In previous studies, high serum ferritin levels were associated with the development of mortality and serious outcomes in COVID-19 [49,77,78]. In this study, high ferritin values in deceased patients were seen as an important risk factor that greatly increased the mortality of the disease. However, the differences in ferritin levels obtained between severely and mildly infected patients in other studies suggested that it may be due to different reasons such as the number of samples included in the study, the infection period of the patients, and secondary bacterial infections.

Increased D-dimer is common in COVID-19 patients and can be attributed to sepsis-induced coagulopathy. In addition, D-dimer may reflect a higher thromboembolic risk in severe cases of COVID-19 [79,80]. In one study, D-dimer levels were found to be significantly higher in severe patients than in non-severe COVID-19 patients and were associated with severe COVID-19 disease [81]. A meta-analysis of 5872 COVID-19 patients found that higher D-dimer concentrations were associated with increased disease severity and mortality in COVID-19 patients [79]. In addition, one study reported that D-dimer (>2.0 mg/L) at admission increased COVID-19 mortality [81]. In another study, it was reported that a reduction in D-dimer levels was observed in recovered patients independent of anticoagulant therapy, while a sustained increase in D-dimer levels may be predictive of a higher risk of thromboembolism and adverse outcomes [82,83]. In a meta-analysis to determine the predictors of COVID-19 severity, Kurniawan et al. [21] found an average of 36.88 mg/L higher CRP, an average of 0.07 ng/mL higher procalcitonin, an average of 0.043 μg/L higher D-dimer, and an average lower -4.58 g/L albumin in severe patients when compare mild patients. In that study, they found AUC for CRP, procalcitonin, LDH, D-dimer, and albumin in the detection of severe COVID-19 patients to be 92%, 89%, 84%, 83%, and 82%, respectively [21]. However, the cut-off values for the features cannot be said to be very successful due to the low sensitivity values of these features and the significant difference between the sensitivity-specificity results of most features. Another study reported that monitoring dynamic variations of D-dimer can be a useful diagnostic tool to predict the prognosis of patients with COVID-19 and peak D-dimer levels may be strongly associated with mortality in patients with COVID-19 [84,85]. In a recent study, Winata and Kurniawan [86] reported a significant increase in D-dimer and fibrinogen degradation products (FDP) in advanced stages of all severe COVID-19 patients with a poor prognosis. Also, they stated that these significant increases in D-Dimer and FDP levels occur due to increased hypoxia in severe and advanced stages COVID-19 conditions, and that increases in these values may be significantly associated with coagulation.

In a meta-analysis study examining the relationship between INR and COVID-19 severity and mortality, it was noted that INR values were significantly increased in dead and severely infected COVID-19 patients [87]. Similarly, previous studies have reported that patients with COVID-19 who died have a higher INR and it was considered a predictor of mortality [85,86]. In this study, D-dimer levels were 2.4 times higher and INR levels were 1.14 times higher in the patients who died from COVID-19 compared to the patients who survived. In addition, in our decision tree, the most important immunological parameter for mortality was INR in patients with ferritin values less than 389.10 μg/L (Fig. 2). The mortality rate was 15.1% in patients with an INR higher than 1,190, while this rate was 0.2% in patients with an INR less than 1190. In addition, D-dimer was found to be the most important immunological parameter for mortality in patients with ferritin values less than 389.10 μg/L and INR less than 1,19 (Fig. 2). Furthermore, the mortality rate was 2.8% in patients with a D-dimer level higher than 1239.40 μg/L, while the mortality rate was zero in patients with a D-dimer level lower than 1239.40 μg/L. According to these results, elevated INR and D-dimer levels increased mortality. In addition, the changes in the ferritin, INR, and D-dimer immunological tests, which were found to be the most effective in the prediction of COVID-19 mortality (Fig. 2), from their values at admission to exit were examined (Fig. 3). Accordingly, the ferritin, INR, and D-dimer values of the patients who died were consistently higher than those of the patients who survived. Another interesting point in our study was that the mortality rate was “zero” in patients with a ferritin value below 389.10 μg/L, an INR value below 1.19, and a D-dimer value below 1239.40 μg/L (Fig. 1). This result was an important indication that this parameter and cut-off values should be included in the scoring systems to be planned for COVID-19 mortality.

Elevated serum CRP levels are key markers of disease progression and a risk factor for mortality in severe COVID-19 patients and have been reported to be indicative of a developing cytokine storm in COVID-19 patients [76,88]. It has been reported that 20 out of 32 studies showed that the risk of poor outcomes was approximately four times higher in COVID-19 patients with high CRP [89]. Also, several studies have found higher levels of procalcitonin in severe COVID-19 patients compared to non-severe patients [31,38,49]. In another study, high procalcitonin levels were found in 85 of 290 patients and they were associated with mortality in COVID-19 patients [89]. In another study, increased procalcitonin values were associated with an approximately 5-fold higher risk of severe SARS-CoV-2 infection [17]. In many studies, they reported that patients who died from COVID-19 had significantly higher levels of CRP, D-dimer, and procalcitonin than surviving patients, and they found that these parameters were associated with the mortality of the disease [4,6,18,31,38,51,88]. Similarly, in many studies, higher CRP, Fibrinogen, D-dimer, troponin, and procalcitonin levels were reported in severely infected and deceased COVID-19 patients compared to living patients [31,38,90,91]. In this study, the CRP (10.70 mg/L fold), fibrinogen (1.10 g/L fold), PT (1.13-sec fold), procalcitonin (22.90 μg/L fold), ESR (2.77 mm/h fold), and troponin (3.35 ng/L fold) values in the non-survivng patients were higher than in the surviving patients. In addition, aPPT (0.88-sec fold) in patients who died was lower than in patients who survived (Table 1). However, in our decision tree (Fig. 2) that was created to determine the immunological parameters on mortality of COVID-19 were not observed a significant effect of fibrinogen, procalcitonin, troponin, and aPPT parameters. According to these results, we can say that INR, D-dimer, and especially ferritin have a more important effect on the prediction of mortality in COVID-19 compared to other immunological parameters.

## 5 Limitations of the study

Our study has potential limitations due to its retrospective nature. In this study, only the immunological parameter values obtained at admission were used to estimate the mortality of the disease. Therefore, how the ferritin values on the days after admission predicted mortality was not investigated. In addition, comorbidity data of the patients were not available. Finally, the patients' pre-COVID-19 ferritin levels were not known, so they could not be compared with post-COVID-19 ferritin values. In addition, it is recommended to test the findings in this study with external datasets in estimating the mortality of COVID-19.

## 6 Conclusion

Identifying risk factors and thresholds for severe and critical COVID-19 patients is important for early and rapid clinical intervention. However, determining the severe infection status of COVID-19 patients using various diagnostic tests and imaging results is costly and time-consuming. In addition, different complications may occur during the procedure. In this case, the patient's health status may be more at risk and the healthcare providers may be under more pressure and tragic situations may occur.

In this study, the characteristics that affect the mortality of severely infected COVID-19 patients and their critical importance levels were determined by the CHAID decision tree, based only on the immunological/inflammatory RBV values measured at hospital admission. The most important features affecting COVID-19 mortality were found to be INR, D-dimer, and especially serum ferritin. The overall accuracy rate of our interpretable decision tree, which was created with only these three features, in classifying COVID-19 groups was found to be 99.0%. In addition, our decision tree correctly identified 99.7% of patients who survived and 92.7% of patients who died.

In this study, 94% of the patients who died from COVID-19 had ferritin levels between 389.10 μg/L and 395.00 μg/L, indicating that this ferritin value range is a critical level for the mortality of COVID-19. In addition, patients with an INR above 1.19 had a much higher mortality rate than the others. Moreover, the mortality rate was 2.8% in patients with D-dimer levels higher than 1239.40 μg/L, while the mortality rate was zero in patients with a D-dimer level lower than 1239.40 μg/L. According to these results, it can be concluded that high INR, D-dimer, and especially ferritin levels significantly increase COVID-19 mortality. We suggest that these features and cut-off values can be used as important biomarkers in the prediction of COVID-19 mortality.

## Ethical approval

Institutional Review Board Statement: The dataset used in this study was collected to be used in various studies in the estimation of the diagnosis, prognosis, and mortality of COVID-19. The necessary permissions for the collected dataset were given by the Ministry of Health of the Republic of Turkey and the Ethics Committee of Erzincan Binali Yıldırım University. This study was conducted following the 1989 Declaration of Helsinki. Erzincan Binali Yıldırım University Human Research Health and Sports Sciences Ethics Committee.

## Informed consent statement

In this study, a dataset including only routine blood values, RTPCR results (positive or negative), and treatment units of the patients was downloaded retrospectively from the information system of our hospital in a digital environment. A new sample was not taken from the patients. There is no information in the dataset that includes identifying characteristics of individuals. It was stated that routine blood values would only be used in academic studies, and written consent was obtained from the institutions for this. In addition, therefore, written informed consent was not administered to every patient.

## Author contribution statement

Mehmet Tahir HUYUT: Conceived and designed the experiments; Performed the experiments; Analyzed and interpreted the data; Contributed reagents, materials, analysis tools or data; Wrote the paper. Zübeyir HUYUT: Analyzed and interpreted the data; Wrote the paper.

## Funding statement

This research did not receive any specific grant from funding agencies in the public, commercial, or not-for-profit sectors.

## Data availability statement

Data associated with this study has been deposited at https://data.mendeley.com/datasets/8hdnzv23 × 7/1.

## Declaration of interest's statement

The authors declare no competing interests.

## References

[1] Zhou C., Chen Y., Ji Y. (2020). Increased serum levels of hepcidin and ferritin are associated with severity of COVID-19. Med. Sci. Mon. Int. Med. J. Exp. Clin. Res..

[2] Huyut M.T., Huyut Z. (2021). Forecasting of Oxidant/Antioxidant levels of COVID-19 patients by using Expert models with biomarkers used in the Diagnosis/Prognosis of COVID-19. Int. Immunopharm..

[3] Chen N., Zhou M., Dong X. (2020). Epidemiological and clinical characteristics of 99 cases of 2019 novel coronavirus pneumonia in Wuhan, China: a descriptive study. Lancet.

[4] Huyut M.T., Huyut Z., İlkbahar F., Mertoğlu C. (2022). What is the impact and efficacy of routine immunological, biochemical and hematological biomarkers as predictors of COVID‐19 mortality?. Int. Immunopharm..

[5] Onur S.T., Altın S., Sokucu S.N. (2021). Could ferritin level be an indicator of COVID‐19 disease mortality?. J. Med. Virol..

[6] Mertoglu C., Huyut M.T., Arslan Y. (2021). How do routine laboratory tests change in coronavirus disease 2019?. Scand. J. Clin. Lab. Invest..

[7] Chalmers J.J., Gómez-Pastora J., Weigand M. (2020). Hyperferritinemia in critically ill COVID-19 patients – is ferritin the product of inflammation or a pathogenic mediator?. Clin. Chim. Acta.

[8] Perricone C., Bartoloni E., Bursi R. (2020). COVID-19 as part of the hyperferritinemic syndromes: the role of iron depletion therapy. Immunol. Res..

[9] Mehta P., McAuley D.F., Brown M. (2020). COVID-19: consider cytokine storm syndromes and immunosuppression. Lancet.

[10] Karakike E., Giamarellos-Bourboulis E.J. (2019). Macrophage activationlike syndrome: a distinct entity leading to early death in sepsis. Front. Immunol..

[11] Cheng L., Li H., Li L. (2020). Ferritin in the coronavirus disease 2019 (COVID-19): a systematic review and meta-analysis. J. Clin. Lab. Anal..

[12] Feld J., Tremblay D., Thibaud S. (2020). Ferritin levels in patients with COVID-19: a poor predictor of mortality and hemophagocytic lymphohistiocytosis. Int J Lab Hematol.

[13] Kernan K.F., Carcillo J.A. (2017). Hyperferritinemia and inflammation. Int. Immunol..

[14] Torti F.M., Torti S.V. (2002). Regulation of ferritin genes and protein. Blood.

[15] Rosário C., Zandman-Goddard G., Meyron-Holtz E.G. (2013). The Hyperferritinemic Syndrome: macrophage activation syndrome, Still's disease, septic shock and catastrophic antiphospholipid syndrome. BMC Med..

[16] Colafrancesco S., Alessandri C., Conti F. (2020). COVID-19 gone bad: a new character in the spectrum of the hyperferritinemic syndrome?. Autoimmun. Rev..

[17] Lippi G., Plebani M., Henry B.M. (2020). Thrombocytopenia is associated with severe coronavirus disease 2019 (COVID‐19) infections: a metaanalysis. Clin. Chim. Acta.

[18] Zhou F., Yu T., Du R. (2020). Clinical course and risk factors for mortality of adult inpatients with COVID-19 in Wuhan, China: a retrospective cohort study. Lancet.

[19] Dimopoulos G., de Mast Q., Markou N. (2020). Favorable Anakinra responses in severe COVID-19 patients with secondary hemophagocytic lymphohistiocytosis. Cell Host Microbe.

[20] Xu X., Han M., Li T. (2020). Effective treatment of severe COVID-19 patients with tocilizumab. Proc. Natl. Acad. Sci. USA.

[21] Kurniawan A., Hariyanto T.I., Japar K.V. (2021). Inflammatory and hematologic markers as predictors of severe outcomes in COVID-19 infection: a systematic review and meta-analysis. AJEM (Am. J. Emerg. Med.).

[22] Hariyanto I.K., Kurniawan A. (2021). Obstructive sleep apnea (OSA) and outcomes from coronavirus disease 2019 (COVID-19) pneumonia: a systematic review and meta-analysis. Sleep Med..

[23] Hariyanto T.I., Siahaan Y.M.T., Ketaren R.J., Hartoyo V. (2021). Epilepsy and the risk of severe coronavirus disease 2019 outcomes: a systematic review, meta-analysis, and meta-regression. Epilepsy Behav..

[24] Kurniawan A., Hariyanto T.I., Putri C., Arisa J., Situmeang R.F.V. (2021). Dementia and outcomes from coronavirus disease 2019 (COVID-19) pneumonia: a systematic review and meta-analysis. Arch. Gerontol. Geriatr..

[25] Gupta V.K., Shukla S.K. (2022). Crime tracking system and people's safety in India using machine learning approaches. International Journal of Modern Research.

[26] Dhiman G., Kumar V. (2017). Spotted hyena optimizer: a novel bio-inspired based metaheuristic technique for engineering applications. Adv. Eng. Software.

[27] Dhiman G., Kumar V. (2018). Emperor penguin optimizer: a bio-inspired algorithm for engineering problems. Knowl. Base Syst..

[28] Huyut M.T., Üstündağ H. (2022). Prediction of diagnosis and prognosis of COVID-19 disease by blood gas parameters using decision trees machine learning model: a retrospective observational study. Med. Gas Res..

[29] Asri H., Mousannif H., Moatassime H.A., Noel T. (2016). Using machine learning algorithms for breast cancer risk prediction and diagnosis. Procedia Comput. Sci..

[30] Chatterjee I. (2021). Patenting machine-learning: review and discussions. International Journal of Modern Research.

[31] Mertoglu C., Huyut M.T., Olmez H. (2022). COVID-19 is more dangerous for older people and its severity is increasing: a case-control study. Med. Gas Res..

[32] Hou H., Zhang B., Huang H. (2020). Using IL-2R/lymphocytes for predicting the clinical progression of patients with COVID-19. Clin. Exp. Immunol..

[33] Ji D., Zhang D., Chen Z. (2020). Clinical characteristics predicting progression of COVID-19. Lancet.

[34] Luchman J.N. (2013).

[35] Magidson J., Bagozzi Richard P. (1994). Advanced Methods of Marketing Research.

[36] Doğanay F., Elkonca F., Seyhan A.U. (2021). Shock index as a predictor of mortality among the Covid-19 patients. Am. J. Emerg. Med..

[37] Kass G.V. (1980). An exploratory technique for investigating large quantities of categorical data. J. Royal Statist. Soc. Series C (Appl. Statistics).

[38] Huyut M.T., İlkbahar F. (2021). The effectiveness of blood routine parameters and some biomarkers as a potential diagnostic tool in the diagnosis and prognosis of covid-19 disease. Int. Immunopharm..

[39] Hancock T.R., Jang T., Li M., Tromp J. (1996). Lower bounds on learning decision lists and trees. Inf. Comput..

[40] McKenzie D.P. (1993). Constructing a minimal diagnostic decision tree. Methods Inf. Med..

[41] Huyut M.T., Kocatürk İ. (2022). The effect of some symptoms and features during the İnfection period on the level of anxiety and depression of adults after recovery from COVID-19. Curr. Psychiatry Res. Rev..

[42] Huyut M.T., Soygüder S. (2022). The multi‐relationship structure between some symptoms and features seen during the new coronavirus 19 infection and the levels of anxiety and depression post‐covid, east. J. Med..

[43] Weiss G., Ganz T. (2019). Goodnough LT: anemia of inflammation. Blood.

[44] Guan W., Ni Z., Hu Y. (2020). Clinical characteristics of coronavirus disease 2019 in China. N. Engl. J. Med..

[45] Li X., Wang L., Yan S. (2020). Clinical characteristics of 25 death cases with COVID-19: a retrospective review of medical records in a single medical center, Wuhan, China. Int. J. Infect. Dis..

[46] Erol A.T., Aşar S., Sabaz M.S. (2021). Risk factors for 28-day mortality among COVID-19 patients in an intensive care unit of a tertiary care center in istanbul. Med J Bakirkoy.

[47] Banerjee A., Ray S., Vorselaars B. (2020). Use of machine learning and artificial intelligence to predict SARS-CoV-2 infection from full blood counts in a population. Int. Immunopharm..

[48] Huyut M.T. (2022). Automatic detection of severely and mildly infected COVID-19 patients with supervised machine learning models. IRBM.

[49] Brinati D., Campagner A., Ferrari D. (2020). Detection of COVID-19 infection from routine blood exams with machine learning: a feasibility study. J. Med. Syst..

[50] Huyut M.T., Velichko A. (2022). Diagnosis and prognosis of COVID-19 disease using routine blood values and LogNNet neural network. Sensors.

[51] Zhang S., Huang S., Liu J. (2021). Identification and validation of prognostic factors in patients with COVID-19: a retrospective study based on artificial intelligence algorithms. J. Intens. Med..

[52] Zhu J.S., Ge P., Jiang C. (2020). Deep-learning artificial intelligence analysis of clinical variables predicts mortality in COVID-19 patients. JACEP.

[53] Alle S., Kanakan A., Siddiqui S. (2022). COVID-19 risk stratification and mortality prediction in hospitalized Indian patients: harnessing clinical data for public health benefits. PLoS One.

[54] Gao Y., Chen L., Chi J. (2021). Development and validation of an online model to predict critical COVID-19 with immune-inflammatory parameters. J. Intensive Care.

[55] Vaishnav P.K., Sharma S., Sharma P. (2021). Analytical review analysis for screening COVID-19 disease. International Journal of Modern Research.

[56] Booth A.L., Abels E., McCaffrey P. (2021). Development of a prognostic model for mortality in COVID-19 infection using machine learning. Mod. Pathol..

[57] Kukar M., Gunčar G., Vovko T. (2021). COVID-19 diagnosis by routine blood tests using machine learning. Sci. Rep..

[58] Mei X., Lee H.C., Diao K. (2020). Artificial intelligence–enabled rapid diagnosis of patients with COVID-19. Nat. Med..

[59] Soares F. (2020).

[60] Soltan A.A., Kouchaki S., Zhu T. (2021). Artificial intelligence driven assessment of routinely collected healthcare data is an effective screening test for COVID-19 in patients presenting to hospital. Lancet Digit Health.

[61] Velichko A., Huyut M.T., Maksim B., Izotov Y., Korzun D. (2022). Machine learning sensors for diagnosis of COVID-19 disease using routine blood values for internet of things application. Sensors.

[62] Huyut M.T., Velichko A., Maksim B. (2022). Detection of risk predictors of COVID-19 mortality with classifier machine learning models operated with routine laboratory biomarkers. Appl. Sci..

[63] Al-Najjar D., Al-Najjar H., Al-Rousan N. (2021). Evaluation of the prediction of CoVID-19 recovered and unrecovered cases using symptoms and patient's meta data based on support vector machine, neural network, CHAID and QUEST Models. Eur. Rev. Med. Pharmacol. Sci..

[64] Guerrero M.D., Vanderloo L.M., Rhodes R.E. (2020). Canadian children's and youth's adherence to the 24-h movement guidelines during the COVID-19 pandemic: a decision tree analysis. J Sport Health Sci.

[65] Feigin E., Levinson T., Wasserman A. (2022). Age-dependent biomarkers for prediction of in-hospital mortality in COVID-19 patients. J. Clin. Med..

[66] Kazawa K., Shimpuku Y., Yoshinaga N. (2022). Characteristics of early-career nurse researchers negatively impacted during the COVID-19 pandemic: a crosssectional study. BMJ Open.

[67] Sacco G., Foucault G., Briere O. (2020). COVID-19 in seniors: findings and lessons from mass screening in a nursing home. Maturitas.

[68] Seddawy A.I.E.L., Mohamed M.H. (2021). The influence of Covid-19 on E-commerce towards online shopping. J. Theor. Appl. Inf. Technol..

[69] Chen G., Wu D., Guo W. (2020). Clinical and immunological features of severe and moderate coronavirus disease 2019. J. Clin. Investig..

[70] Gandini O., Criniti A., Ballesio L. (2020). Serum ferritin is an independent risk factor for acute respiratory distress syndrome in COVID-19. J. Infect..

[71] Gao Yd, Ding M., Dong X. (2021). Risk factors for severe and critically ill COVID-19 patients: a review. Allergy.

[72] Dahan S., Segal G., Katz I. (2020). Ferritin as a marker of severity in COVID-19 patients: a fatal correlation. Isr. Med. Assoc. J..

[73] Taneri P.E., Gómez-Ochoa S.A., Llanaj E. (2020). Anemia and iron metabolism in COVID-19: a systematic review and meta-analysis. Eur. J. Epidemiol..

[74] Huang I., Pranata R., Lim M.A. (2020). C-reactive protein, procalcitonin, D-dimer, and ferritin in severe coronavirus disease-2019: a meta-analysis. Ther. Adv. Respir. Dis..

[75] Ruscitti P., Giacomelli R. (2020). Ferritin and severe COVID-19, from clinical observations to pathogenic implications and therapeutic perspectives. Isr. Med. Assoc. J..

[76] Perricone C., Bartoloni E., Bursi R. (2020). COVID-19 as part of the hyperferritinemic syndromes: the role of iron depletion therapy. Immunol. Res..

[77] Azkur A.K., Akdis M., Azkur D. (2020). Immune response to SARSCoV- 2 and mechanisms of immunopathological changes in COVID-19. Allergy.

[78] Riggioni C., Comberiati P., Giovannini M. (2020). A compendium answering 150 questions on COVID-19 and SARS-CoV-2. Allergy.

[79] Danwang C., Endomba F.T., Nkeck J.R. (2020). A meta-analysis of potential biomarkers associated with severity of coronavirus disease 2019 (COVID-19). Biomark Res.

[80] Bompard F., Monnier H., Saab I. (2020). Pulmonary embolism in patients with COVID-19 pneumonia. Eur. Respir. J..

[81] Yu H.H., Qin C., Chen M. (2020). D-dimer level is associated with the severity of COVID-19. Thromb. Res..

[82] Lala A., Johnson K.W., Januzzi J.L. (2020). Prevalence and impact of myocardial injury in patients hospitalized with COVID-19 infection. J. Am. Coll. Cardiol..

[83] Yao Y., Cao J., Wang Q. (2020). D-dimer as a biomarker for disease severity and mortality in COVID-19 patients: a case control study. J Intensive Care.

[84] Liu Y., Du X., Chen J. (2020). Neutrophil-to-lymphocyte ratio as an independent risk factor for mortality in hospitalized patients with COVID-19. J. Infect..

[85] Ye W., Chen G., Li X. (2020). Dynamic changes of D-dimer and neutrophil- lymphocyte count ratio as prognostic biomarkers in COVID-19. Respir. Res..

[86] Winata S., Kurniawan A. (2021). Coagulopathy in COVID-19: a systematic review. Via Medici.

[87] Zinellu A., Paliogiannis P., Carru C. (2021). INR and COVID-19 severity and mortality: a systematic review with meta-analysis and meta-regression. Adv. Med. Sci..

[88] Zhang J.J., Cao Yy, Tan G. (2021). Clinical, radiological, and laboratory characteristics and risk factors for severity and mortality of 289 hospitalized COVID-19 patients. Allergy.

[89] Malik P., Patel U., Mehta D. (2021). Biomarkers and outcomes of COVID-19 hospitalisations: systematic review and meta-analysis. BMJ Evid Based Med.

[90] Mikami T., Miyashita H., Yamada T. (2020). Risk factors for mortality in patients with COVID-19 in New York city. J. Gen. Intern. Med..

[91] Khinda J., Janjua N.Z., Cheng S. (2021). Association between markers of immune response at hospital admission and COVID‐19 disease severity and mortality: a meta‐analysis and meta‐regression. J. Med. Virol..

